# Human cardiac stem cells inhibit lymphocyte proliferation through paracrine mechanisms that correlate with indoleamine 2,3-dioxygenase induction and activity

**DOI:** 10.1186/s13287-018-1010-2

**Published:** 2018-10-25

**Authors:** Maria J Sebastião, Ramón Menta, Margarida Serra, Itziar Palacios, Paula M Alves, Belén Sanchez, Olga DelaRosa, Wilfried Dalemans, Eleuterio Lombardo, Patrícia Gomes-Alves

**Affiliations:** 1 Animal Cell Technology Unit, iBET, Instituto de Biologia Experimental e Tecnológica, Oeiras, Portugal; 2 ITQB-NOVA, Instituto de Tecnologia Química e Biológica António Xavier, Universidade Nova de Lisboa, Oeiras, Portugal; 3 Coretherapix, S.L.U. (TiGenix Group), Tres Cantos, Spain; 4 TiGenix SAU, Tres Cantos, Madrid, Spain; 5 TiGenix NV, Leuven, Belgium

**Keywords:** Cardiac stem/progenitor cells, Allogeneic stem cell therapy, Tryptophan metabolism, Immunosuppression, T lymphocytes

## Abstract

Transplantation of allogeneic human cardiac/stem progenitor cells (hCSCs) is currently being tested in several phase I/II clinical trials as a novel and promising therapy for restoration of myocardial tissue function in acute myocardial infarction (AMI) patients. Previous findings demonstrate that these cells have an immune suppressive profile interacting with different populations from the immune system, resulting in overall attenuation of myocardial inflammation. However, transplanted hCSCs are still recognized and cleared from the injured site, impairing long retention times in the tissue that could translate into a higher clinical benefit.

In this work, through modeling allogeneic hCSC/T lymphocyte interaction in vitro by direct contact, transwell inserts, and hCSC conditioned medium, our results demonstrate that hCSCs exert an immune-suppressive effect on T lymphocyte proliferation not only through the previously described cell contact-dependent programmed cell death-1 (PD1)/programmed death ligand-1 (PDL-1) axis but also through a paracrine mechanism associated with indoleamine 2,3-dioxygenase (IDO) enzyme-mediated tryptophan metabolism. Such findings constitute a step forward in better understanding the mechanisms of action of transplanted hCSCs in allogeneic settings.

**Electronic supplementary material:**

The online version of this article (10.1186/s13287-018-1010-2) contains supplementary material, which is available to authorized users.

## Introduction

Human cardiac/stem progenitor cell (hCSC) transplantation is becoming a promising therapy for acute myocardial infarction (AMI), one of the most prevalent causes of death worldwide [1]. CSCs are considered by several authors as the preferred candidate cell source for AMI patients, mainly due to their function in the heart, well-documented paracrine regenerative properties [2, 3], and the success of transplantation studies in myocardial infarction animal models [4, 5].

Such success in preclinical stages has led to a rapid translation to the clinic, and several phase I and II clinical trials using hCSCs as an autologous therapy have emerged (e.g., SCIPIO and CADUCEUS trials). Autologous transplantation, although carrying lower immunogenicity risks, holds several limitations. The quality of the cells might be compromised by patient age and comorbidities [3, 6, 7], as well as logistic, economic, and time-constraints issues. To overcome such limitations, in the last years the field has been moving towards allogeneic CSC sources (e.g., the ALLSTAR and CAREMI trials).

Clinical trials have demonstrated physiological improvements and increases in viable tissue and heart functional outcome [8]. However, an obstacle still preventing CSCs from meeting their full clinical potential and to provide evident clinical benefit over a standard-of-care is their limited retention and engraftment in the heart [9, 10], a problem aggravated in the allogeneic setting [11, 12].

An immune response is triggered upon AMI that, although essential for proper tissue remodeling and stabilization, carries unwanted inflammatory-mediated damage [13] and, in the case of cell therapy approaches, might be also involved in the elimination of injected cells. Effective activation of T cells, one of the main mediators of inflammatory damage upon AMI, requires simultaneous engagement of the T-cell receptor (TCR) and CD28 receptor. TCR binds to antigens present in major histocompatibility complex (MHC; human leukocyte antigens (HLA) in humans) and CD28 receptor binds to B7 (CD80/CD86) costimulatory molecules. hCSCs express HLA class I molecules that attract killer cytotoxic T cells, but very low levels of HLA class II molecules that stimulate antibody-producing B cells, and do not express the costimulatory molecules CD80/CD86 [14], therefore presenting a weak immunogenic profile. Besides depicting the immune phenotype of hCSCs, several studies have shed some light on how hCSCs interact with monocytes [15], natural killer cells [16], and T lymphocytes [14, 17]. Similar to other studies with mesenchymal stem cells (MSCs) [18–21], all the referred studies with hCSCs suggest that the immunologic behavior of cells might be linked to their therapeutic effects rather than eliciting deleterious immune reactions, with an immunomodulatory effect resulting in attenuation of inflammation. Furthermore, programmed death ligand 1 (PDL-1)-mediated cell-cell interaction has been identified as one of the main mechanisms for the immunomodulatory properties of hCSCs, promoting stimulation of regulatory T cells and subsequent inhibition of T lymphocyte activation and proliferation [14].

Besides direct cell-cell interactions, paracrine immunomodulatory effects based on extracellular vesicles have been described for hCSCs [17]. Tryptophan (Trp) metabolism through the enzymatic activity of indoleamine 2,3-dioxygenase (IDO) has been described as a key immunosuppressive mechanism for human adipose-derived mesenchymal stem cells (hASCs) [22–24], a cell type already used in several allogeneic cell transplantation studies [25].

With the aim of better understanding the immunomodulatory mechanisms of hCSCs in an allogeneic setting, we further investigated the capacity of hCSCs to inhibit the proliferation of T lymphocytes in vitro. Taken together, our results add to knowledge on the tolerogenic immune behavior of hCSCs, showing that hCSC-mediated immune modulation is not dependent exclusively on the PDL-1/ programmed cell death-1 (PD-1) pathway axis, but also via Trp degradation by IDO enzyme action. Such findings open new avenues for designing novel hCSC allogeneic transplantation therapies for AMI patients, including strategies to promote a higher hCSC engraftment and longer residence time in the tissue.

## Results

**Fig. 1:**
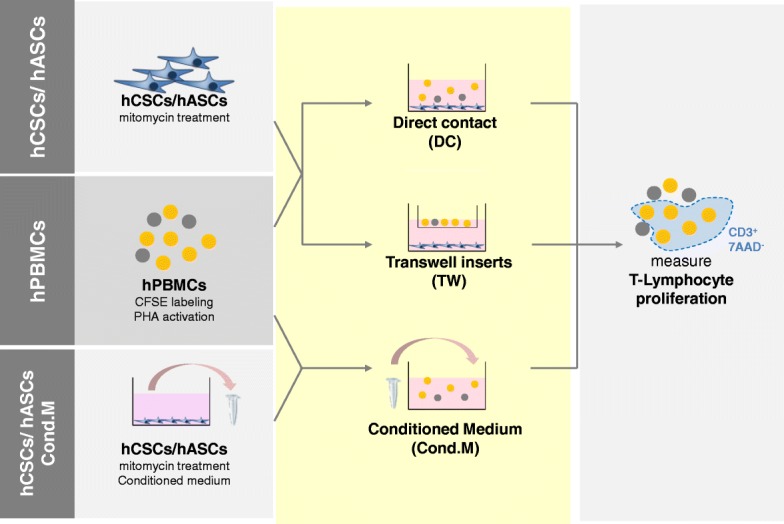
Schematic representation of immunomodulatory assay experiments. Carboxyfluorescein succinimidyl ester (CFSE)-labeled and phytohemagglutinin (PHA)-activated human peripheral blood mononuclear cells (hPBMCs) were cultured with mitomycin-treated human cardiac/stem progenitor cells (hCSCs)/human adipose-derived mesenchymal stem cell (hASCs) either in direct contact (DC), in a transwell support (TW), or in contact with mitomycin-C-treated hCSC/hASC conditioned medium (Cond.M.). Viable T lymphocyte proliferation was accessed by CFSE labeling of the CD3^+^ 7AAD^–^ hPBMC population

The immunomodulatory experimental design is illustrated in Fig. 1. Briefly, stimulated human peripheral blood mononuclear cells (hPBMCs) were cultured in the presence of mitomycin C-treated hCSCs or hASCs. Cultures were performed in culture well plates in: 1) direct contact (DC); 2) using transwell (TW) inserts to allow exchange of soluble factors but separation of both cell types; and 3) using hCSC conditioned medium. hASCs were used as a positive control for T cell proliferation inhibition via IDO. See Additional file 1 for detailed materials and methods.

### IDO expression is induced in hCSCs in response to interferon (IFN)-γ

**Fig. 2:**
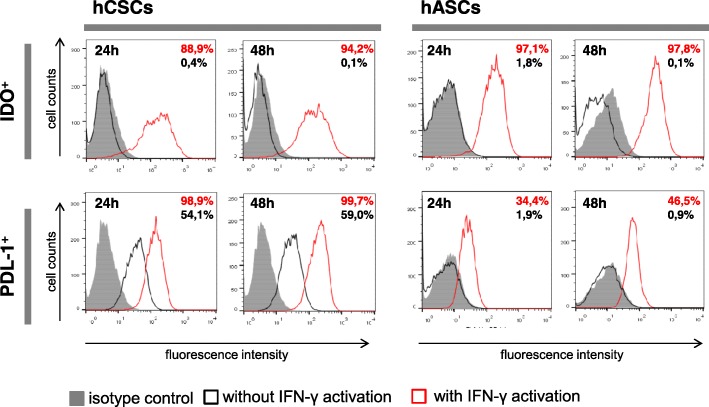
Human cardiac/stem progenitor cells (hCSCs) display a favorable immune-suppressive phenotype. Representative expression of the immune relevant molecules indoleamine 2,3-dioxygenase (IDO) and programmed death ligand 1 (PDL-1) after 24 and 48 h in untreated (black line histograms) and interferon (IFN)-γ-activated (red line histograms) cells against isotype controls (gray-filled histograms). The percentages (%) of positive cells are indicated. Human adipose-derived mesenchymal stem cells (hASCs) were used as a positive control for IDO expression. hCSC results are shown for donor hCPC8. Other donors presented similar results

We first characterized the hCSC expression of the immune-relevant molecules PDL-1 and IDO with and without IFN-γ stimulation. While hASCs only express PDL-1 when activated, hCSCs express PDL-1 constitutively, and activation with IFN-γ further upregulates its expression (Fig. 2). These results agree with data previously reported by Lauden et al. [14]. Neither hCSCs nor hASCs displayed any constitutive expression of IDO but both cell types displayed significant expression upon stimulation with IFN-γ (Fig. 2).

### hCSCs impair activated T lymphocyte proliferation

Following the characterization of a favorable immune-suppressive phenotype of hCSCs, we then examined whether these cells were capable of inhibiting T lymphocyte proliferation in an allogeneic setting. Stimulated hPBMCs were cultured in direct contact with hCSCs at different ratios.

**Fig. 3:**
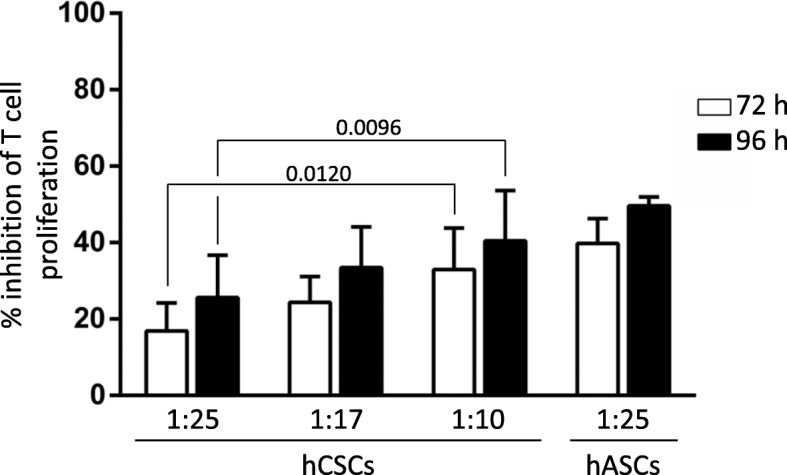
Human cardiac/stem progenitor cells (hCSCs) inhibit T lymphocyte proliferation in a time- and hCSC concentration-dependent manner. CFSE-labeled hPBMCs were stimulated with PHA and cultured alone or in direct contact with hCSCs (ratios 1:10, 1:17, and 1:25 hCSCs:hPBMCs). After 72 h (white bars) and 96 h (black bars), proliferation of the viable population of CD3-viable T lymphocytes (CD3^+^/7AAD^–^) was assayed by loss of CFSE staining. Percentage of inhibition of proliferation was determined using FSC Express software against proliferation of activated hPBMCs alone. Human adipose-derived mesenchymal stem cells (hASCs) were used as a positive control for T cell proliferation inhibition (ratio 1:25 hASCs:hPBMCs). Adjusted *p* values are shown

As previously reported [14], hCSCs exert an immune suppressive role by inhibiting T lymphocyte proliferation. hCSCs have a significant suppressive effect in T lymphocyte proliferation in a dose-dependent manner, although to a lesser extent compared with hASCs (Fig. 3). Although no significant difference was seen between the time points, there is a tendency for higher proliferation inhibition at 96 h versus 72 h of incubation (Fig. 3) in all ratios analyzed, suggesting that this effect may also be time dependent.

### hCSCs immunomodulatory capacity can occur in the absence of cell-cell contact

To evaluate the importance of IDO enzyme and Trp metabolism in the immunosuppressive capacity of hCSCs, we carried out T lymphocyte proliferation assays in which hCSCs were not in direct contact (DC) with hPBMCs and therefore cannot exert their immunomodulatory activity through the PDL-1/PD1 axis.

**Fig. 4:**
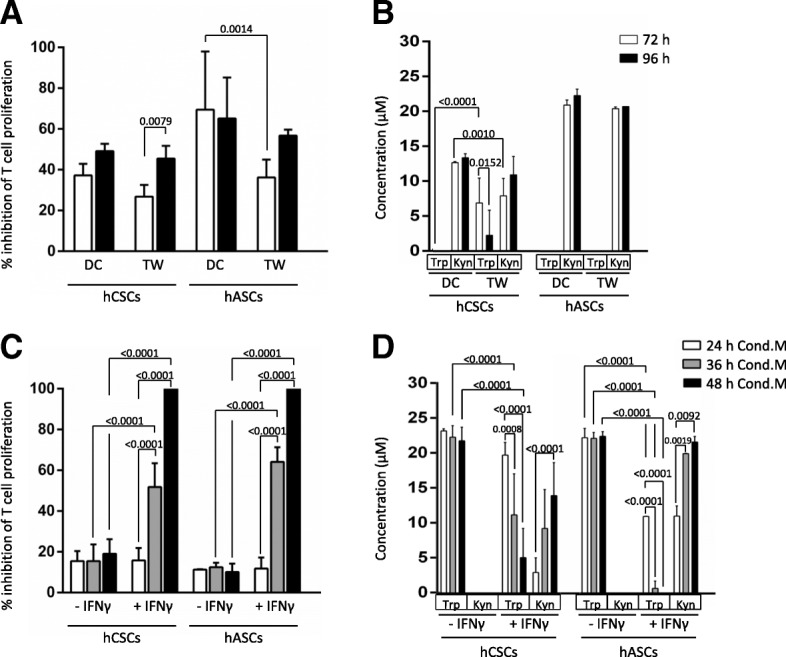
Human cardiac/stem progenitor cells (hCSCs) inhibit T lymphocyte proliferation via a paracrine mechanism. **a** CFSE-labeled hPBMCs were stimulated with PHA and cultured alone, in direct contact (DC), or in a transwell setting (TW) with hCSCs (ratio 1:10 hCSCs:hPBMCs). **b** Concentrations of tryptophan (Trp) and kynurenine (Kyn) were determined by HPLC in the supernatants. **c** CFSE-labeled hPBMCs were stimulated with PHA and cultured alone or in conditioned medium (Cond.M.) from hCSCs cultures activated or not with interferon (IFN)-γ. Conditioned media were generated for 24 h (white bars), 36 h (grey bars), and 48h (black bars). **d** Concentrations of Trp and Kyn were determined by HPLC in the conditioned media. Proliferation of the viable population of CD3 T lymphocytes (CD3^+^/7AAD^–^) was assayed by loss of CFSE staining after 72 h (white bars) and 96 h (black bars) for TW and DC experiments (**a**) and after 96 h for Cond.M. experiments (**c**). Percentage of cells per generation and percentage of inhibition of proliferation was determined using FSC Express software against proliferation of activated hPBMCs alone. Human adipose-derived mesenchymal stem cells (hASCs) were used as a positive control for T cell proliferation inhibition (ratio 1:25 hASCs:hPBMCs). Adjusted *p* values are shown

We carried out hCSC-hPBMC coculture under transwell conditions (TW), allowing paracrine interaction between the cell types. At 72 h of incubation, although slightly lower when compared with DC, hCSCs do exert a significant suppressive effect on T lymphocyte proliferation under TW conditions. Moreover, such a difference between TW and DC conditions was lost after 96 h of incubation (Fig. 4a).

Trp metabolism was also assessed by measuring Trp and kynurenine (Kyn; a Trp metabolite described as cytotoxic for T lymphocytes [26]) concentrations in the conditioned medium. As shown in Fig. 4b, Trp is fully depleted at 72 h under the DC condition, and in the TW setting it is also significantly diminished when compared with stimulated hPBMCs alone. Furthermore, the accumulation of Kyn occurred in both experimental setups (Fig. 4b).

Besides the TW experiments, hCSC conditioned medium was generated for 24 h, 36 h, and 48 h using control and IFN-γ-stimulated hCSCs. Similar to the hASC control, hCSC-derived conditioned medium significantly inhibited T lymphocyte proliferation, with a significant increase in IFN-γ-stimulated cells (51.79 ± 11.67 % versus 15.49 ± 8.10% with 36-h conditioned medium; 100 ± 0.00 % versus 19.01 ± 7.22% with 48-h conditioned medium; Fig. 4c). Moreover, conditioned medium from longer IFN-γ-stimulated hCSC cultures prompted higher inhibition of T lymphocyte proliferation (Fig. 4c). Such findings are also in accordance with the Trp and Kyn measurements, where Trp is gradually depleted and Kyn gradually accumulates in the supernatant of hCSC cultures (Fig. 4d).

## Discussion

Allogeneic hCSC-based therapies continue to be explored as an alternative for AMI patients. However, hCSC regenerative medicine approaches have yet to show an evident and robust clinical benefit over standard-of-care. Although described as having a positive immunomodulatory role rather than eliciting further inflammation [14–17], one of the main challenges to be addressed in hCSC transplantation-based therapies is the rapid elimination of the injected cells. A better knowledge of the immunological properties of hCSCs is therefore paramount in developing strategies to increase the retention time of the cells in the myocardium that would consequently increase their regenerative benefits.

Similar to what is described for MSCs [18–21], hCSCs have been described as having an immune-suppressive profile. Several studies show that hCSCs in allogeneic settings have an anti-inflammatory profile by modulation of natural killer cell cytokine secretion and cytotoxicity [16], by modulation of monocytes, macrophages, and dendritic cells [15], and by activation of T regulatory lymphocytes with subsequent inhibition of T lymphocyte proliferation via PDL-1/PD1 direct cell communication [14], as well as via extracellular vesicle-mediated paracrine communication [17].

In this work, we assayed the immunomodulatory capacity of hCSCs in an inflammatory setting by IFN-γ activation and explored the hypothesis of paracrine IDO-mediated T lymphocyte proliferation inhibition.

IFN-γ is highly expressed under inflammatory settings (such as after AMI). This proinflammatory pleiotropic cytokine is produced primarily by the host T lymphocytes in response to antigen recognition and has also been shown to induce the expression of immune-relevant molecules in several stem cell populations, including neural stem cells [27] and MSCs [24, 28]. IFN-γ was shown to cause hCSCs to upregulate the expression of both class I and II HLA molecules [14], which indicates that their administration into an inflammatory myocardium environment probably increases their recognition by host T lymphocytes. On the other hand, IFN-γ supplementation has also previously been shown to upregulate hCSC expression of PDL-1, resulting in a stronger hCSC immune-suppressive profile [14].

IFN-γ activation has also been correlated with increased IDO expression in human MSCs, including hASCs, which in turn has been shown to be a key enzyme involved in the immunomodulatory capacity of these cells [22, 29]. IDO suppresses T lymphocyte proliferation and promotes T lymphocyte death through degradation of Trp, an essential amino acid required for cell proliferation and subsequent accumulation of cytotoxic Trp metabolites (including Kyn) [26, 30]. IFN-γ also causes the activation of tryptophanyl-transfer RNA synthetase (WRS; an aminoacyl synthetase that incorporates Trp into proteins) in IDO-expressing cells, which has been postulated to be a compensatory mechanism allowing IDO-expressing cells to better cope with Trp depletion [31].

In our study, we show that IFN-γ activation is correlated with an increase in hCSC PDL-1 and IDO expression. Although with an overall weaker immune-suppressive profile when compared with hASCs, we also showed that hCSCs are able to inhibit T lymphocyte proliferation in a time-and hCSC cell concentration-dependent manner when in direct coculture.

Moreover, we showed Trp depletion and Kyn accumulation in activated hCSC conditioned medium. Concordantly, stimulated hCSC conditioned medium showed a superior antiproliferative effect when compared with unstimulated hCSC conditioned medium, suggesting a relevant role of IDO-mediated Trp metabolism in the immunomodulatory paracrine effect of hCSCs.

We also showed no significant differences between DC and TW in terms of T lymphocyte proliferation, suggesting that paracrine-mediated effects are a central mechanism of action for hCSCs, as previously demonstrated with Sca1^+^ hCSCs [17].

These findings provide evidence that, although playing a role in the process, PDL-1 mediated T-regulatory cell modulation is not the exclusive nor the central mechanism involved in T lymphocyte proliferation inhibition, at least under our experimental conditions. This finding further supports the prominent paracrine-based beneficial CSC activities in the host tissue.

In this work, we provide evidence of Trp metabolism as a novel mechanism involved in the hCSC-mediated T lymphocyte proliferation suppression properties. We also hypothesize that, similar to what is already described for hASCs [22–24], IDO is the main player in hCSC Trp metabolism in inflammation settings in vitro. Complementary studies to further test this hypothesis should include analysis of WRS expression, Trp supplementation, and IDO inhibition experiments to further validate IDO Trp metabolism as a main player in hCSC immunomodulatory properties in the host tissue.

## Additional file

Additional file 1: Detailed material and methods: a full description of material and methods including cell isolation and culture, immunomodulatory assays, and statistical analysis. (DOCX 26 kb)

## Abbreviations

AMI: Acute myocardial infarction
CSC: Cardiac/stem progenitor cell
DC: Direct contact
hASC: Human adipose-derived mesenchymal stem cell
hCSC: Human cardiac/stem progenitor cell
HLA: Human leukocyte antigen
hPBMC: Human peripheral blood mononuclear cell
IDO: Indoleamine 2,3-dioxygenase
IFN: Interferon
Kyn: Kynurenine
MHC: Major histocompatibility complex
MSC: Mesenchymal stem cell
PD1: Programmed cell death-1
PDL-1: Programmed death ligand 1
TCR: T-cell receptor
Trp: Tryptophan
TW: Transwell
WRS: Tryptophanyl-transfer RNA synthetase
