# Supplementary material for: Human cardiac stem cells inhibit lymphocyte proliferation through paracrine mechanisms that correlate with indoleamine 2,3-dioxygenase induction and activity
Source: Stem Cell Res Ther. 2018 Oct 25;9:290. doi: 10.1186/s13287-018-1010-2 (PMC6202863; doi:10.1186/s13287-018-1010-2)
Supplement: Supplementary file 1 — Detailed material and methods: a full description of material and methods including cell isolation and culture, immunomodulatory assays, and statistical analysis. (DOCX 26 kb) [file 13287_2018_1010_MOESM1_ESM.docx]

**Additional File 1**

**Material and Methods**

# hASCs isolation and culture

Human ASCs were isolated from adipose tissue lipoaspirates obtained from healthy adult donors as described elsewhere [1]. hASCs specific surface markers were verified by flow cytometry: hASCs were positive for HLA-I, CD90, and CD105, and negative for HLA-II, CD40, CD80, CD86, and CD34. The following primary antibody dilutions were used: CD105 (1:50), remaining (1:10). A total of ten thousand events were acquired using a FACSCalibur (BD Biosciences). Percentage of positive cells was calculated using the FSC-express software.

Cells were cultured at 37°C in humidified incubators (5% CO_2_, 3% O_2_) in DMEM medium containing 10% FBS. Medium was replaced every 7 days. Cells were subcultured when the culture achieved about 90% confluency using Trypsin-EDTA 0,05% for 5 minutes at 37°C. Prior to immunomodulatory experiments, hASCs were treated with mitomycin-C (20 μg/mL) at 37°C for 30 min. All cell culture reagents were purchased from Gibco, Life Technologies unless otherwise stated. Cells were used at a population doubling level of 14.

# hCSC isolation and culture

Human CSCs were obtained from human right atria appendage myocardial tissue, isolated and characterized as described elsewhere [2]. Cells were cultured at 37°C in humidified incubators (5% CO_2_, 3% O_2_) in Expansion Medium (ExpM, composed by DMEM:F12: Neurobasal medium (1:1), supplemented with 1% penicillin streptomycin, 10% Fetal Bovine Serum embryonic stem cell-qualified, N2 Supplement (1X), B27 Supplement (1X), 0.9 mM L-Glutamine, 50 μM β-Mercaptoethanol (Sigma), Insulin Transferrin Selenium (0.5X), 10 ng/mL bFGF, 20 ng/mL EGF-I and 30 ng/mL IGF-II (Prepotech), all percentages in (v/v). Medium was replaced by 50% every 3 days. Cells were subcultured when the culture achieved about 80% confluency using Trypsin-EDTA 0,05% for 5 minutes at 37°C. Prior to immunomodulatory experiments, hCSCs were treated with mitomycin-C (20 μg/mL) at 37°C for 30 min. All cell culture reagents were purchased from Gibco, Life Technologies unless otherwise stated.

hCSCs from 3 different donors were used in this study: hCSC-35 (Female, 17 years), hCSC-40 (Female, 79 years) and hCSC-48 (Female, 45 years). Cells were used at passages 4–6.

# Isolation of hPBMCs

Blood samples were provided by the National Transfusion Centre of the Comunidad Autónoma (Madrid, Spain). Human peripheral blood mononuclear cells (hPBMCs) were isolated from the buffy coats by density centrifugation gradient using Ficollplaque Plus (GE Healthcare Biosciences AB), in accordance with manufacturer instructions. Blood samples from 3 different donors were used in the experiments.

# CFSE labelling

Proliferation of hPBMCs was accessed with carboxyfluorescein succinimidyl ester (CFSE) labeling as previously described [3]. Briefly, cells were washed, resuspended in a 20 µM CFSE solution (10^7^ hPBMCs per 200 µL of solution) and incubated under constant shaking at 37°C for 10 min. The reaction was stopped by slowly adding ice-cold RPMI medium supplemented with 10% FBS (v/v). Cells were then cultured overnight, and one aliquot was used to set up and control the FL-1 voltage for CFSE.

# IDO and PDL-1 expression

hCSCs were seeded in culture well plates and either left unstimulated or stimulated with IFN-γ (3 ng/mL). After 24 h and 48 h of culture, cells were trypsinized and stained with anti-PDL-1 antibody (2.5:100, ThermoFisher Scientific) and anti-IDO antibody (4:100, ThermoFisher Scientific) and isotype control IgG (2.5:100, BD Biosciences) using the kit for intracellular staining from eBioscience, following the manufacturer’s instructions. A total of ten thousand events were acquired using a FACSCalibur (BD Biosciences). hASCs were used as a positive control IDO expression.

# Generation of conditioned supernatants

hASCs (0.2x10^6^) and hCSCs (0.5x10^6^) were seeded per well in 6 well plates, treated with mitomycin-C (20 μg/mL, at 37°C for 30 min) and cultured in RPMI medium supplemented with 10% FBS (v/v) with or without IFN-γ stimulation (3 ng/mL) for 24, 36 and 48 hours. Supernatants were collected, centrifuged to remove dead cells and debris and stored at -20°C until further use for immunomodulatory assays.

# Immunomodulatory assays

CFSE-labeled hPBMCs were stimulated with phytohemagglutinin (PHA, 1 µg/mL) (Sigma-Aldrich) and cultured in the presence of mitomycin C-treated hCSCs and hASCs at three different ratios (hCSC/hASCs:hPBMC ratios 1:10, 1:17 and 1:25) in RPMI + 10% FBS (v/v).

Cultures were performed in culture well plates in i) direct contact (DC); ii) using transwells (TW) inserts with a 0.4 mm pore size (Corning) to allow exchange of soluble factors but separation of both cell types; and iii) using hCSCs conditioned medium (Cond.M). Cells were cultured at 37°C in RPMI medium supplemented with 10% FBS (v/v), in humidified incubators (5% CO2, 3% O2). hASCs were used as a positive control for T cell proliferation inhibition via IDO (ratio 1:25 hASCs:hPBMCs). Parallel control experiments with hPBMCs alone were also performed. After 72 and 96 h (DC and TW experiments) or after 96 h of culture (Cond.M experiments), CFSE-labeled hPBMCs were harvested, labeled with 7-AAD and anti-CD3 antibody, and cell proliferation of the CD3^+^/ 7-AAD^-^ population (viable CD3 T lymphocytes) was evaluated by flow cytometry (FACSCalibur, BD Biosciences) according to loss of CFSE signal. A total of ten thousand events were acquired using a FACSCalibur (BD Biosciences). Division index and percentage of inhibition of proliferation were calculated using the FSC-express software.

# IDO activity

IDO enzyme activity was measured by determining both Trp and kynurenine (Kyn) concentrations in conditioned medium. About 200 µL of conditioned medium was added to 50 mM phosphate buffer and 25 mL of 2 M trichloroacetic acid. After centrifugation for 10 min at 15,600 g, the supernatant was collected and analyzed by high-performance liquid chromatography (HPLC), Waters 717plus Autosampler using a Waters 1515 Isocratic Pump, and a Waters 2487 Dual Absorbance Detector. Separation was performed using a C18 4.6 50mm column,(Teknokroma Analítica S.A.) in 10 min runs with 40 mM sodium citrate (pH 5.0), 1% acetonitrile as mobile phase at a flow rate of 1 mL/min. Trp and Kyn were detected at an absorbance of 280 and 360 nm, respectively.

# Statistical analysis

Statistical analyses were performed with GraphPad Prism6 (GraphPad Software Inc.). All data are shown as mean with standard deviation. Data was analyzed by One Way ANOVA Tukey test. P-values below 0.05 were considered significant. Three different hCSC biological replicates (from 3 different donors) were used.

**References**

1. Mancheño-Corvo P, Menta R, del Río B, Franquesa M, Ramírez C, Hoogduijn MJ, et al. T Lymphocyte Prestimulation Impairs in a Time-Dependent Manner the Capacity of Adipose Mesenchymal Stem Cells to Inhibit Proliferation: Role of Interferon γ, Poly I:C, and Tryptophan Metabolism in Restoring Adipose Mesenchymal Stem Cell Inhibitory Effect. Stem Cells Dev. 2015;24:2158–70.

2. Lauden L, Boukouaci W, Borlado LR, López IP, Sepúlveda P, Tamouza R, et al. Allogenicity of human cardiac stem/progenitor cells orchestrated by programmed death ligand 1. Circ Res. 2013;112:451–64.

3. DelaRosa O, Lombardo E, Beraza A, Mancheno-Corvo P, Ramirez C, Menta R, et al. Requirement of IFN-gamma-mediated indoleamine 2,3-dioxygenase expression in the modulation of lymphocyte proliferation by human adipose-derived stem cells. Tissue Eng Part A . 2009;15:2795–806.
